# Exchange-bias and magnetic anisotropy fields in core–shell ferrite nanoparticles

**DOI:** 10.1038/s41598-021-84843-0

**Published:** 2021-03-09

**Authors:** F. G. Silva, J. Depeyrot, Yu. L. Raikher, V. I. Stepanov, I. S. Poperechny, R. Aquino, G. Ballon, J. Geshev, E. Dubois, R. Perzynski

**Affiliations:** 1 Instituto de Física, Universidade de Brasília, Caixa Postal 04455, Brasília, 70919-970 Brazil; 2 Sorbonne Université, CNRS, PHENIX UMR 8234, 75005 Paris, France; 3 Faculdade UnB Planaltina, Universidade de Brasília, Planaltina (DF), 73345-010 Brazil; 4 Institute of Continuous Media Mechanics, Ural Branch of RAS, Perm, 614068 Russia; 5 Institute of Natural Sciences and Mathematics, Ural Federal University, Ekaterinburg, 620083 Russia; 6 Department of Phase Transitions Physics, Perm State National Research University, Perm, 614990 Russia; 7 CNRS-LNCMI, 31400 Toulouse, France; 8 Instituto de Fisica, UFRGS, Porto Alegre, RS 91501-970 Brazil

**Keywords:** Materials science, Nanoscale materials, Magnetic properties and materials

## Abstract

Exchange bias properties of MnFe_2_O_4_@$\gamma$–Fe_2_O_3_ core–shell nanoparticles are investigated. The measured field and temperature dependencies of the magnetization point out a well-ordered ferrimagnetic core surrounded by a layer with spin glass-like arrangement. Quasi-static SQUID magnetization measurements are presented along with high-amplitude pulse ones and are cross-analyzed by comparison against ferromagnetic resonance experiments at 9 GHz. These measurements allow one to discern three types of magnetic anisotropies affecting the dynamics of the magnetic moment of the well-ordered ferrimagnetic NP’s core viz. the easy-axis (uniaxial) anisotropy, the unidirectional exchange-bias anisotropy and the rotatable anisotropy. The uniaxial anisotropy originates from the structural core–shell interface. The unidirectional exchange-bias anisotropy is associated with the spin-coupling at the ferrimagnetic/spin glass-like interface; it is observable only at low temperatures after a field-cooling process. The rotatable anisotropy is caused by partially-pinned spins at the core/shell interface; it manifests itself as an intrinsic field always parallel to the external applied magnetic field. The whole set of experimental results is interpreted in the framework of superparamagnetic theory, i.e., essentially taking into account the effect of thermal fluctuations on the magnetic moment of the particle core. In particular, it is found that the rotatable anisotropy of our system is of a uniaxial type.

## Introduction

The exchange bias (EB) effect had been discovered about 50 years ago at ferromagnet–antiferromagnet (FM/AFM) interfaces in fine Co/CoO nanoparticles^1^. In contemporary understanding, see a comprehensive review^2^, the origin of EB is associated with the spin clusters whose magnetic state is defined by both their intrinsic anisotropy and the exchange coupling (pinning) to the phases on both sides of the FM/AFM interface. Due to that, a part of those spins does not follow the bulk FM magnetization when it is driven by an applied field^3^. As it has turned out, the EB effect at an FM/AFM border is observed as a common feature of magnetic nanoparticles^2,4–6^ as well as of thin films and superlattices^7–9^. Moreover, essentially the same manifestations are inherent to virtually any interface between the structures with different spin ordering: ferrimagnets (FiM), spin glasses, etc.^2^. In comparison to films, the EB effects per unit surface area are more pronounced in nanoparticles as their sub- and on-surface layers occupy the greater volumic fraction. There the surface spins, being frustrated due to the geometry, impurities, etc., self-organize in complex patterns resembling distorted AFM or rather spin glass-like (SGL) structures^10,11^. The high specific strength of the occurring EB effects makes magnetic nanoparticles prospective for such high-tech applications as data storage, spintronics, nanomedicine, etc.^12^.

The core–shell (CS) particles, like the bi-layer films, have both an interface and a surface, and so are prone to complex EB phenomena^2,13–15^. In majority, the conventional theoretical models assuming the core to be a highly-ordered (bulk-like) magnetic object, treat the shell as just a ferrite layer with a wide spread of local anisotropy axes^16^. It is assumed that at enhanced temperatures the orientational distribution of those axes is tunable by an external field. By cooling under field, this distribution is fixed and becomes the source of unidirectional EB anisotropy which affects the magnetic moment of the core. A clear example of that behavior is reported in Ref.^17^ for Fe@$\gamma$–Fe_2_O_3_ CS nanoparticles. The akin effect is proven to cause intra- and interparticle EB in ultrasmall MnFe_2_O4@$\gamma$–Fe_2_O_3_ and CoFe_2_O_4_@$\gamma$–Fe_2_O_3_ nanoparticles^18,19^. It results in an intrinsic field ${\varvec{H}}_{\mathrm{EB}}$ stemming from the pinned spins at the FiM/SGL interface^20^, which manifests itself by a shift $H_{\mathrm{ex}}$ of field-cooled (FC) hysteresis magnetization loops^1^. Very recently, the magnetic saturation criteria and their relation with both anisotropy and EB fields have been investigated in such systems^21^.

The above-mentioned theoretical schemes, whatever useful, do not reflect in full the diversity of experimentally observed effects of the EB origin. In particular, comparative analysis of the quasistatic and magnetodynamic (ferromagnetic resonance) measurements points out that in CS particles, as in multilayer films, the exchange coupling, along with customary EB anisotropy, causes another type of anisotropy^22,23^. The latter is known as rotatable anisotropy (RA)^24^, and is unambiguously detected in ferromagnetic resonance (FMR) experiments where it manifests itself as an additional internal field ${\varvec{H}}_{\mathrm{RA}}$ that readily follows the direction of the imposed magnetizing field ${\varvec{H}}$. Contrarily to the origin and properties of unidirectional EB anisotropy, the RA effect is much less studied, especially in nanoparticles of CS type. We can name but few theoretical works^25,26^ and a rather short list of experimental ones^18,27^ on that subject. Moreover, up to now, when considering the RA, there is yet no consensus even on its symmetry: whether it is unidirectional^28,29^, as the standard EB one, or bidirectional, i.e., uniaxial^3,22^. Meanwhile, the existence of the RA as such complies in full with the general concept that the interface spin clusters together with the spin layers adjacent to the surfaces are responsible for a plethora of strong and observable magnetic effects^2^.

In this paper, taking as test objects the samples consisting of MnFe_2_O_4_@$\gamma$–Fe_2_O_3_ CS nanoparticles dispersed in a carrier, we show that the temperature dependence of anisotropy fields ($H_{\mathrm{EB}}$ and $H_{\mathrm{RA}}$) is a key issue for distinguishing the interfacial magnetic anisotropies of CS nanoparticles. By combining dc magnetization and FMR measurement data we could separate the unidirectional (EB) and uniaxial (RA) contributions. To account for the experimental evidence, we propose a superparamagnetic model and consider three contributions to the NPs anisotropy, namely the uniaxial core anisotropy, the EB unidirectional contribution and the rotatable anisotropy. It enables one to analyze the symmetry of the RA effect and to assess (at least, by order of magnitude) the pertinent magnetic characteristics of CS particles.

## Results

Measurements are performed on series of three different NPs, S1, S2, S3, of different mean size (see Table 1 and “SI”), either in powder or individually dispersed in a carrier, which is fluid at room temperature (see “Methods”).

**Table 1:** Core/shell and magnetic characteristics of NPs samples.

Sample	$d_{0}$ (nm)	*s*	$\phi _{s}$/$\phi _{p}$	$t_{sh}$ (nm)	$M_{c} (0)$ (kA/m)	$K_{s}$ (J/m^2^)	$H_{A}$ (kA/m)
S1	7	0.3	0.25	0.47	515	$2.5 \times 10^{5}$	60
S2	3.6	0.4	0.56	0.54	375	$3.0 \times 10^{5}$	200
S3	2.8	0.4	0.56	0.42	200	$3.6 \times 10^{5}$	530

$d_{0}$ is the median diameter, *s* the polydispersity index, ${\phi _{s}}/{\phi _{p}}$ the volume fraction of the maghemite shell, $t_{sh}$ its thickness, $M_{c} (0)$ the core magnetization at $T=0$ K, $K_{s}$ the surface anisotropy constant of the maghemite shell and $H_{A}$ the anisotropy field.

### Magnetization measurements—FiM/SGL interface of core shell ferrite nanoparticles

**Figure 1:**
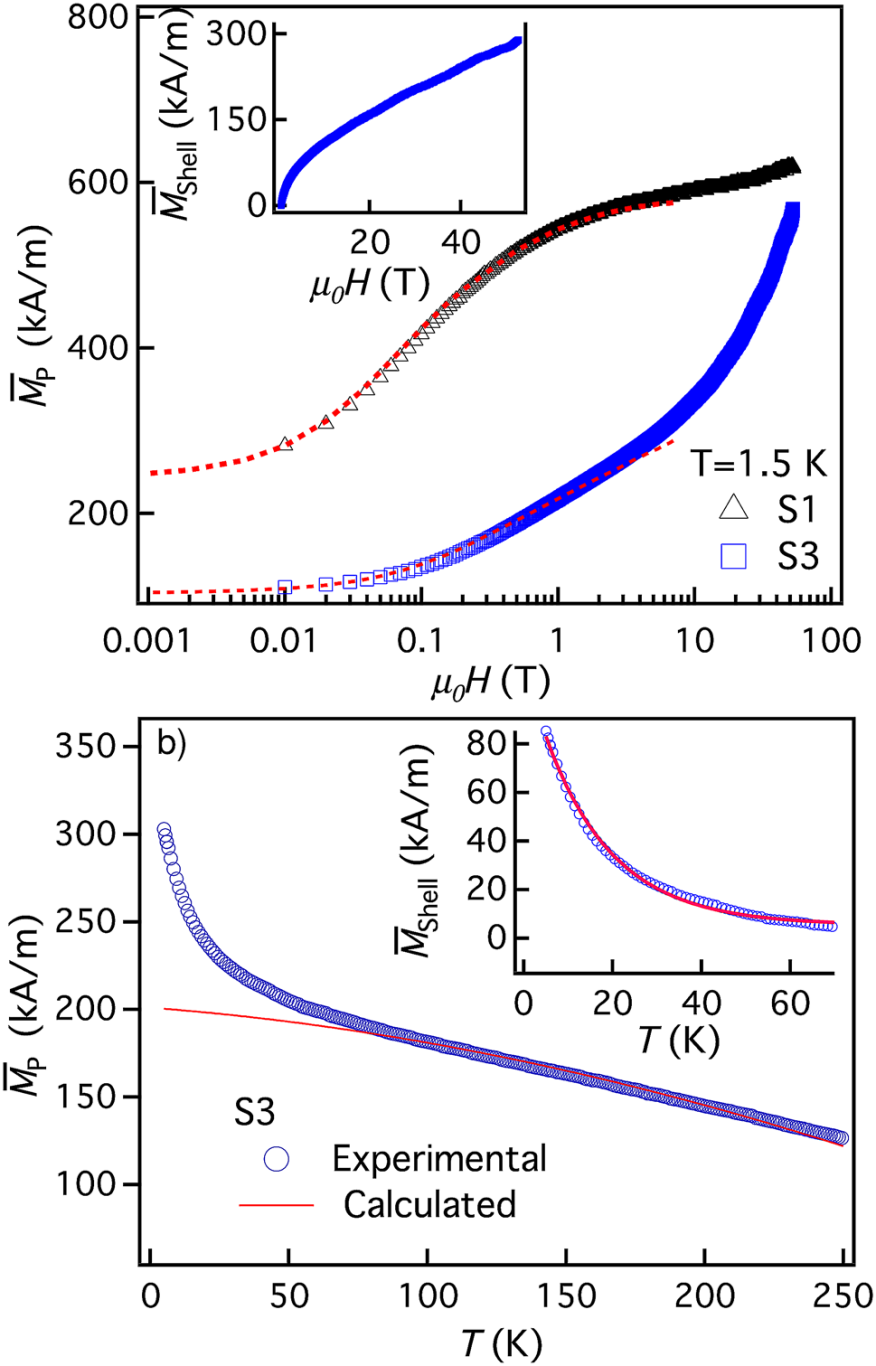
(**a**) Reduced ZFC magnetization ${{\overline{M}}}_p$ of samples S1 and S3 under field $\mu _0H$ going down from 52 T; dotted line shows the lower field SQUID calibration. Inset: extracted shell magnetization ${{\overline{M}}}_{sh}$ of sample S3, see “SI”. (**b**) Temperature dependence of ${{\overline{M}}}_p$ for sample S3 (open circles) at 5 T; full line renders the core contribution ${{\overline{M}}}_c(T)$ (see the text).

Descending branches of the magnetic hysteresis loops in samples S1 and S3 are presented in Fig. 1a. The overall NP’s magnetization ${{\overline{M}}}_p$ is size-dependent and does not saturate even at 52 T; the smaller the NPs the stronger the effect. The considerable change of slope of the curve characterizing S1 stems from the non-linearity of the magnetization. Under the field decrease it gradually passes from the saturation regime (small slope) to nearly linear regime (much greater slope).

Then, as in Refs.^30,31^, the overall NP magnetization is considered as the sum of core and shell contributions. We thus extract the field variations of the average shell magnetization by subtracting from the overall NP magnetization the core contribution calculated following the model detailed in “SI” (Sects. 2 and 3). One obtains ${{\overline{M}}}_{sh}(52\,\text {T})\simeq 300$ kA/m for all the three samples. Typical variations of the average shell magnetization are presented at the inset in Fig. 1a for sample S3. It indicates that ${{\overline{M}}}_{sh}$ is yet far from saturation even at 52 T.

Figure 1b displays the temperature dependence of the overall NP magnetization recorded with high temperature resolution at ${{\mu }}_{0}H=5$ T. Following Ref.^31^, at high temperature the smooth variations are attributed to the thermal dependence of the FiM core contribution caused by thermal excitation of spin waves and the low temperature upturn is associated with the freezing of surface spins in a disordered SGL structure. By considering two additive contributions, one can extract the corresponding thermal variations of the shell magnetization ${\overline{M}}_{sh}$ (see for details in Sects. 2 and 3 of “SI”). Briefly, we calculate here, differently from Ref.^31^, the temperature dependence of the core magnetization in a more rigorous way taking into account the NPs polydispersity. The measurement data interpreted with the aid of this approach are presented in Fig. 1, where the full line renders the core magnetization. The temperature dependence of the deduced shell contribution ${{\overline{M}}}_{sh}$ (see inset) is well adjusted as in Ref.^31^ with $\mathrm {exp}(- {T}/15)$ with *T* in Kelvins.

### FMR measurements—under-field textured vs non-textured samples

In liquid dispersions (ferrofluids), the NPs are mechanically free, so that their easy axes orient themselves in compliance with the applied field. Taking a dilute ferrofluid and freezing it under zero field, one fixes the isotropic distribution of the particle axes. If freezing is performed under a constant field $H_f$, then the NP easy axes texture is quenched possessing the degree of orientation that depends on the field strength $H_f$, on the freezing temperature $T_f$ of the dispersion, and on the anisotropy energy as well as on the nature and size of the NPs.

**Figure 2:**
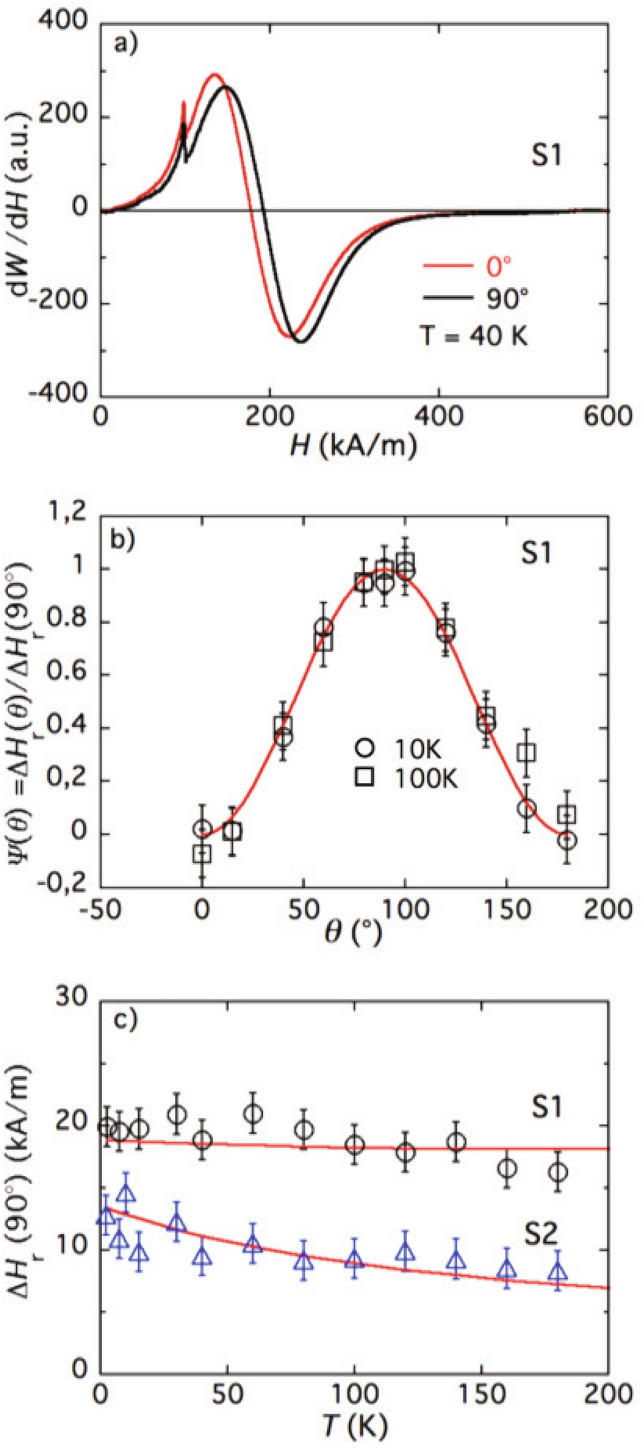
(**a**) Typical FMR spectra of sample S1. As in Ref.^27^, the first small peak at $\sim 100$ kA/m is due to residual impurities in the glass micropipe containing the ferrofluid sample. (**b**) Angular dependence of the resonance field for textured sample S1 at $T=10$ and 100 K; full line is ($1-\cos ^{2}\theta$); (**c**) Maximum shift $\Delta H_r(90^\circ )$ of the FMR field as a function of temperature for samples S1 and S2; full lines are calculated using Eq. (2).

Results of X band FMR measurements, collected on diluted NP’s dispersions in various configurations (textured or not, that have been field-cooled under $H_{cool}=800$ kA/m or not) are presented in the following—see “Methods” for the details of experimental method. The global analysis is based on a superparamagnetic theoretical model of single nanoparticles, describing their energy of anisotropy as the sum of three contributions experienced by the core magnetic moment, a uniaxial one proportional to the NP’s surface, a unidirectional exchange bias (only observed after a field-cooling process) and a uniaxial rotatable anisotropy which is “thawing” as temperature increases. Let us first look at the core contribution. Figure 2a shows the spectra recorded at 40 K on sample S1 for two values of the angle $\theta$ between the magnetizing field and the axis of orientational texture. When $\theta$ is varied from $0^\circ$ to $90^\circ$, the lines display a typical increase of the resonance field $H_r(\theta )$ and such a behavior is observed for all the three samples in the whole studied temperature range. From angular variations of the spectra, one is able to identify the symmetry and strength of the particle anisotropy^27^. An example is shown in Fig. 2b for sample S1 at both 10 K and 100 K where the experimental values of the reduced shift $\psi (\theta )$ of the resonance field:

1 $$\begin{aligned} \Psi (\theta )=\frac{\left[ H_r(\theta )-H_r(0^\circ )\right] }{\left[ H_r(90^\circ )-H_r(0^\circ )\right] }= \frac{\Delta H_r(\theta )}{\Delta H_r(90^\circ )}, \end{aligned}$$

are presented as a function of $\theta$. These variations fairly well confirm the easy-axis type of the anisotropy of sample S1 which follows the ($1 - {\text{ cos }}^2 \theta$) dependence of $\Delta H_r(\theta )$, see Ref.^32^. As inferred in Refs.^29^ and^27^, in chemically homogeneous particles this anisotropy field ${\varvec{H}}_{\textsc {A}}$ is imposed on the core magnetic moment by outer surface spins. In CS case, the structure border between different ferrites may also affect the symmetry of anisotropy^19,33^. Similar observations are obtained for CuFe_2_O_4_@$\gamma$–Fe_2_O_3_ and NiFe_2_O_4_@$\gamma$–Fe_2_O_3_ core–shell nanoparticles with a mean NP diameter slightly larger than that of sample S1 (data not shown).

The uniaxial anisotropy field in an assembly of particles whose axes ${\varvec{n}}$ are all parallel, at $T=0\,{\text {K}}$ would equal $\tfrac{2}{3}\Delta H_r(90^\circ )$. In a nanoparticle dispersion frozen at a finite temperature under a field, whatever high, the alignment of ${\varvec{n}}$’s is never perfect. At $T\ne 0\,\text {K}$, thermal fluctuations cause diminution of $\Delta H_r(90^\circ )$ with both temperature and particle size. Figure 2c, where samples S1 ($d_0=7.0$ nm) and S2 ($d_0=3.6$ nm) are compared, evidences that point.

The full lines adjustments of Fig. 2c are obtained with a superparamagnetic model of an assembly of uniaxial particles orientationally textured under field ${\varvec{H}}_f$. We assume that the (cubic) bulk magnetic anisotropy of the particle core is negligible in comparison with its uniaxial surface anisotropy whose axis ${\varvec{n}}$ is rigidly fixed with respect to the particle body. This anisotropy is characterized by energy $E_{\textsc {A}}=\pi K_sd^2$ with $K_s$ being the surface anisotropy density, see Sect. 4 of “SI”. In the adopted model, the difference between FMR fields corresponding to the configurations with magnetizing field ${\varvec{H}}$ either perpendicular ($\theta =90^\circ$) or parallel ($\theta =0^\circ$) to ${\varvec{H}}_f$, is:

2 $$\begin{aligned} \Delta H_r(90^\circ )=\frac{18K_s}{\mu _0M_cd}\frac{L_2(\xi _L)}{L_1(\xi _L)}\, L_2(\xi _f)S_2(\sigma _f); \end{aligned}$$

here $\xi _L=\mu _0\mu \omega /\gamma kT$ with $\omega$ being the exciting frequency (constant in field-sweep FMR experiments) and $\gamma$ the gyromagnetic ratio, so that $\omega /\gamma$ is the nominal resonance field of Larmor precession. The Langevin factors $L_1$ and $L_2$, see Sect. 4 of “SI”, account for the “internal” superparamagnetism of the particle: fluctuation-induced deviations of the magnetic moment from the direction of the easy axis. The orientation order parameter of easy axes in Eq. (2) is defined as:

3 $$\begin{aligned} S_2(\sigma _f)\!=\!\frac{3}{2}\left[ \frac{d}{d\sigma _f}\ln R(\sigma _f)-\frac{1}{3}\right] , \; \quad \quad R(\sigma )\!\!=\!\!\int _0^1\exp (\sigma x^2)\,dx. \end{aligned}$$

The other notations in (2) are $\xi _f=\mu _0\mu H_f/kT_f$ and $\sigma _f=E_{\textsc {A}}/kT_f$. The details of these calculations are presented in Sect. 4 of “SI”.

The results of that model when it is applied to the measurements on samples S1 and S2, are shown in Fig. 2c and demonstrate good agreement. The parameters used for fitting are listed in Table 1. Note that the range of $K_s$’s fairly well matches the one found for maghemite NPs^29^ and, in turn, agrees with Néel’s predictions. The fields $H_{\textsc {A}}$ resulting from this uniaxial anisotropy, extrapolated to 0 K from the FMR data adjustments, are $\simeq 60$ and 200 kA/m for S1 and S2, respectively.

**Figure 3:**
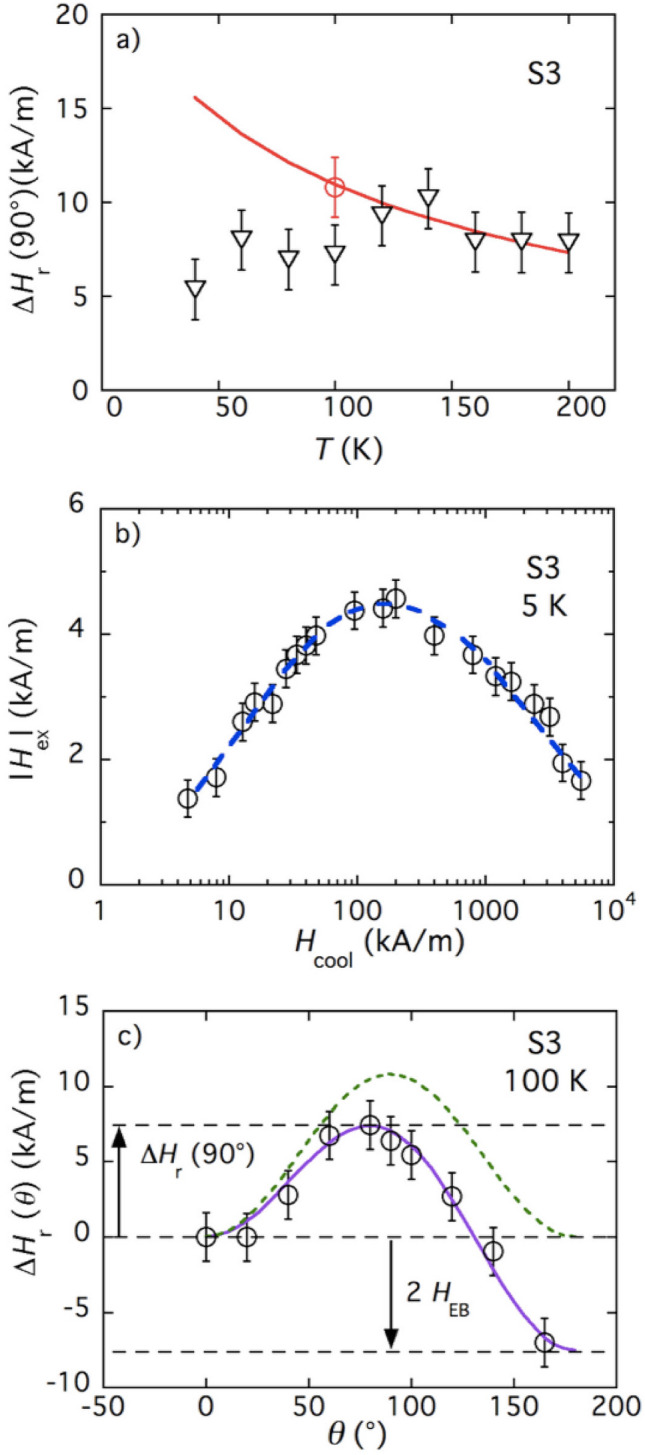
(**a**) Triangular symbols: thermal variations of $\Delta H_r(90^\circ )$, difference between FC-FMR fields obtained for $\theta =90^\circ$ and $\theta =0^\circ$), for textured sample S3; full line: adjustment of $\Delta H_r(90^\circ )$ in the absence of ${\varvec{H}}_{\textsc {EB}}$ (see Eq. 2); red open circle: uniaxial contribution to $\Delta H_r(90^\circ )$ deduced from the fit of (**c**). (**b**) Cooling-field dependence of the shift $H_{ex}$ of FC quasistatic hysteresis loops for non-textured sample S3. (**c**) Angular dependence of the resonance field shift $\Delta H_r(\theta )$ for textured sample S3 at $T=100$ K; dashed line represents the angular variations of the uniaxial contribution; full line corresponds to the fit of the data by Eq. (4), see text for details.

The same model applied to FMR data of sample S3 only fits at the highest experimental *T*’s. The best fit performed with this model is represented by the full line in Fig. 3a. The results are obtained with $M_c(0)$ and $K_s$ values given in Table 1, which corresponds to $H_{\textsc {A}}\simeq 530$ kA/m (extrapolated to 0 K) in fair agreement with $H_{\textsc {A}}\simeq 400$ kA/m assessed from first ZFC magnetization curve at 5 K in Ref.^19^. The model only account for thermal variations above 120 K typically. We will show in the following that this surprising behavior can be enlightened since sample S3 mostly comprises extra-small NPs which exhibit unidirectional exchange bias anisotropy.

## Discussion

Although the size distribution is close to that of sample S2, the in-field Mössbauer measurements of sample S3 evidence strong internal disorder, which is not observed in S2^34,35^. Moreover, sample S3 is distinguished by acquiring a strong exchange bias after taking it in a solidified non-textured state and then field-cooling it to low temperatures^18,19^. These exchange-bias properties progressively disappear as temperature is increased^19^. The negative shift $H_{ex}$ of the quasistatic magnetization loops of the FC sample S3 (with the probing field ${\varvec{H}}$ parallel to the cooling field ${\varvec{H}}_{cool}$) is the signature of unidirectional anisotropy field $H_{\textsc {EB}}$ originating from the FiM/SGL interface^19^. The effect of NPs interaction and of the nature of the core ferrite on $H_{ex}$ has been studied in Refs.^18,19^. The dependence of $H_{ex}$ on the cooling field is extracted from FC hysteresis loops and displayed in Fig. 3b (data from Ref.^19^). The presence of a maximum is attributed to the depinning threshold above which part of the spins in the SGL layer aligns with the external magnetic field^36^. Notably, the position of the maximum is proportional to the anisotropy field $H_{\textsc {A}}$^19^. We remark that the decrease of $H_{ex}$ might be attributed to the increasing number of FiM ordered spins (SGL layer thinning) as the cooling field strength grows. AC/DC susceptibility measurements in CS systems at different applied fields have been proposed in Refs.^17,37^ in order to understand the different responses of the core and shell layers to the strength of the applied magnetic field and to correlate them to the EB effect. We plan to perform such measurements in a near future to complement the here-presented rf probing, enlightening the complex interplay between the different disordered spins at the surface of the particles.

**Figure 4:**
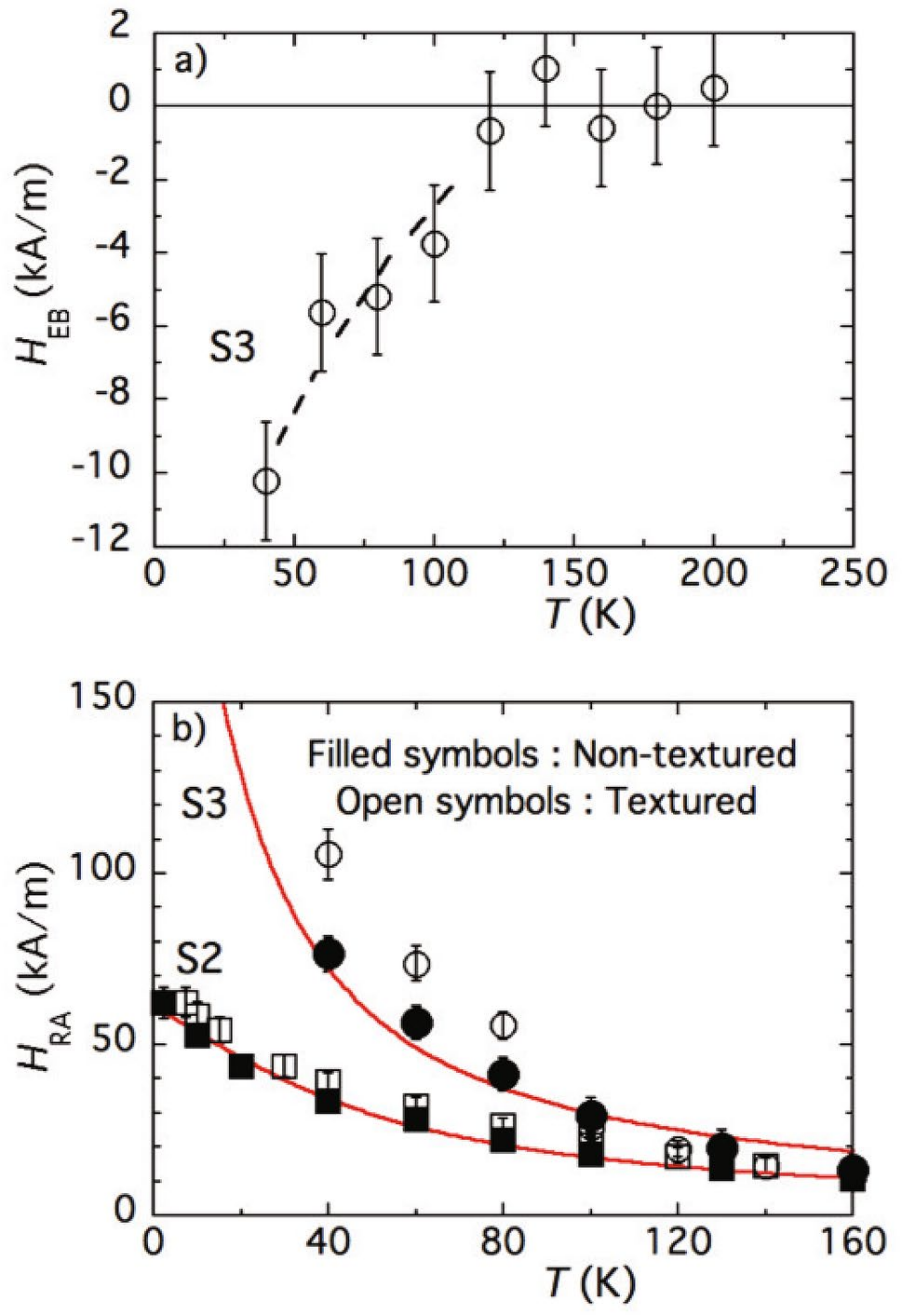
(**a**) Temperature dependence of the exchange bias field $H_{\textsc {EB}}$ deduced from the anisotropy of the FMR field for sample S3. (**b**) Thermal variation of $H_{\textsc {RA}}(T)$ for samples S2 and S3; full lines represent the adjustments of ZFC measurements by Eq. (5).

In such a context, the two temperature regimes of Fig. 3a—below and above 120 K—clearly distinguished on the thermal variations of $\Delta H_r(90^\circ )$ of sample S3 should be associated with the presence/absence of $H_{\textsc {EB}}$, see Eq. (17) of “SI” with $\vartheta =\vartheta _c$. At $T \ge 120$ K, NPs behave as just uniaxial particles with $H_{\textsc {EB}}=0$ and without extra surface contribution to magnetization, see Fig. 1b. At $T=100$ K, the uniaxial symmetry of the angular dependence of $H_r(\theta )$ is broken as shown in Fig. 3c due to the presence of exchange bias $H_{\textsc {EB}}$, see, for example, Eq. (17) in “SI”. Thus we describe the angular dependence of $\Delta H_r(\theta )$ by a two-term expression

4 $$\begin{aligned} A(1-\cos ^2\theta )+B(1-\cos \theta ) \end{aligned}$$

with coefficients $A=10.8\pm 1.6$ kA/m and $B=-3.8\pm 1.6$ kA/m obtained from fitting. As seen, coefficient *A* is the amplitude of the uniaxial angular contribution to $\Delta H_r(\theta )$ in the absence of exchange bias (red circular mark at Fig. 3a). The angular dependence of this uniaxial contribution is the dashed line in Fig. 3c. Coefficient *B* could be presented in the form $\tfrac{1}{2}\left[ H_r(180^\circ )-H_r(0^\circ )\right]$, and it renders the effect of unidirectional anisotropy that produces a negative exchange bias field $H_{\textsc {EB}}$, see Ref.^23^. This contribution, indistinguishable at 120 K, becomes observable at $T=100$ K and below, thus breaking the high-temperature uniaxial (bidirectional) symmetry of $H_r(\theta )$. We note that the value of *B*, as derived from FMR experiments, is of the same sign and order of magnitude as $H_{ex}$ observed at 5 K in quasistatic magnetization measurements on the non-textured FC samples prepared under the same cooling field: $H_{cool}=1.6\times 10^3$ kA/m, see Fig. 3b. To estimate $H_{\textsc {EB}}$ in the whole experimental range of temperature, one should take the difference between the experimental data and the unixial fit (solid line) in Fig. 3a. The result is presented in Fig. 4a; we note that for sample S3 the FC-FMR measurements cannot be performed below 40 K due to experimental limitations. However, it is seen that between 40 and 120 K, as temperature grows, $H_{\textsc {EB}}$ decreases in absolute value to virtual vanishing. This behavior is very probably strongly affected by relaxation and training effects, i.e., by the time lapse between the initial field-cooling and the actual measurement^19^.

The third contribution that we can extract from the model detailed in “SI” is attributed to rotatable anisotropy (RA) whose origin in core–shell particles is associated with a wide spread of exchange parameters in the SGL layer. Due to it, the pinning of magnetic structure in the FC regime never occurs in full. Besides a well pinned fraction, there remain some regions but weakly exchange-anchored to the main part of the layer. Those regions interact with each other forming an arrangement similar to that of AFM domain structure^22^. Because of the small size of the clusters, the activation energy for their correlated reorientation is low, which allows this quasi-domain structure to easily adjust to the changes of the direction of external field. Therefore, in CS particles, along with the customary (quenched) EB anisotropy whose axis is insensitive to external fields of moderate strength, there is present another component of the exchange origin whose axis is indeed rotatable. In other words, the field ${\varvec{H}}_{\textsc {RA}}$ acts on the particle magnetic moment as if its direction is always aligned with the applied one.

Due to the mentioned lability of the spin cluster structure, the effect of $H_{\textsc {RA}}$ does not manifest itself in quasistatic experiments. However, it readily reveals itself under FMR conditions, i.e., at 9 GHz and higher^27,28^. Indeed, a weak rf probing field induces small deviations of the core magnetic moment ${\varvec{\mu }}$ from its equilibrium orientation that is defined by the static (magnetizing) field ${\varvec{H}}$. The latter, in turn, defines the equilibrium direction of ${\varvec{H}}_{\textsc {RA}}$, which acts on ${\varvec{\mu }}$ as any other anisotropy field. The imposed rf field ($\lesssim 100$ A/m) does not affect ${\varvec{H}}_{\textsc {RA}}$ (at least in the linear approximation) since the energy of the induced perturbations is yet much weaker than, albeit low, thresholds necessary to move the RA axis. On the other hand, the magnetizing field $\sim 260$ kA/m used in our FMR experiments turns out to be more than sufficient to do that: our data evidences that under rotating a solidified sample, the direction of ${\varvec{H}}_{\textsc {RA}}$ readily follows ${\varvec{H}}$. In below, we address the temperature dependence of ${\varvec{H}}_{\textsc {RA}}$.

We base our approach on the following. First, at the temperatures above the blocking point, superparamagnetism has a decisive effect on ${\varvec{\mu }}$. Second, in FMR measurements $H_{\textsc {RA}}$ is always lower in strength than ${\varvec{H}}$ and might be considered as a perturbation. Third, as the NPs are small (a few nm), then following Ref.^15^, one may assume that the main role in $H_{\textsc {RA}}$ formation belongs to the interior spins of the NP’s shell and not to the interface. This points out just one source of RA, which affects the whole particle, core and shell altogether.

The FMR problem for a particle, whose magnetic moment moves under joint action of the external field and combination of several anisotropy fields being also affected by thermal fluctuations of arbitrary strength, can be formulated and solved in the manner similar to that of Ref.^38^. Upon doing that, see “SI”, one obtains the superparamagnetic contribution to the temperature dependence of the RA field in the form

5 $$\begin{aligned} H_{\textsc {RA}}(T)=H_{\textsc {RA}}^{(0)}\frac{L_2(\xi _L)}{L_1(\xi _L)}=\frac{\omega }{\gamma }-\frac{1}{3}\left[ 2H_r(90^\circ )+H_r(0^\circ )\right] , \end{aligned}$$

where $H_{\textsc {RA}}^{(0)}$ is the value of this field at $T=0$. This formula, although looking specified for measurements on orientationally textured samples, works as well for isotropic ones, where its right-hand side reduces to $\omega /\gamma -H_r$. As expected for RA, expression (5) comes out angular-independent. Its low-temperature limit ($\xi _L\rightarrow \infty$) gives $H_{\textsc {RA}}(0)=H_{\textsc {RA}}^{(0)}$, whereas for high temperatures ($\xi _L\ll 1$) formula (5) renders

6 $$\begin{aligned} H_{\textsc {RA}}(T)=\frac{2}{5} H_{\textsc {RA}}^{(0)}\cdot \xi _L\propto T^{-1}. \end{aligned}$$

As Eq. (6) shows, with temperature growth $H_{\textsc {RA}}(T)$ gradually falls down from the value that it has at $T=0$, RA “thaws” as *T* increases. Physically, this is due to the enhancement of orientational diffusion of the particle moment.

We remark that in the literature, one can find very few data on RA properties of core–shell nanoparticles and less so on the temperature dependence of $H_{\textsc {RA}}$. Besides, according to Ref.^16^ the minutes of preparation and post-preparation treatment affect the results substantially. However, the “thawing” of RA comes out to be its inherent qualitative feature, see points in Fig. 4b presenting the thermal variations of $H_{\textsc {RA}}$ deduced from measurements on both orientationally textured and non-textured samples S2 and S3. Our model (Eq. 5) (solid lines in Fig. 4b) infers that the main source of $H_{\textsc {RA}}$ falling down with temperature, is superparamagnetism.

In fact, a much more general conclusion can be driven. From the theoretical considerations given in Ref.^38^ and “SI”, it follows that the fluctuation-induced thermal behavior of the anisotropy field determined from FMR measurements depends on the symmetry of the corresponding energy term as $L_j(\xi _L)/L_1(\xi _L)$, where *j* is the tensor rank of the term. To see that, it suffices to compare the two possible contributions associated with $E_{\textsc {RA}}$ proposed in Ref.^25^: unidirectional versus uniaxial. Indeed, superparamagnetism does not affect unidirectional ($j=1$) anisotropy, while for uniaxial ($j=2$) one, it induces temperature diminution, see (Eq. 5). Therefore, just a qualitative analysis of the temperature dependence of $H_{\textsc {RA}}$ of our CS particles identifies their rotatable anisotropy as being uniaxial. The rank $j=4$ is inherent to cubic magnetic anisotropy. An example of pertinent calculation could be found in Ref.^38^, where it is shown that the temperature fall of the cubic anisotropy contribution in the FMR field is much steeper than that for the uniaxial case.

## Conclusions

In MnFe_2_O_4_@$\gamma$–Fe_2_O_3_ core–shell nanoparticles, by a set of dc and rf measurements, we have separated three magnetic anisotropy contributions, namely uniaxial, unidirectional and rotatable. The two last terms are associated with the exchange bias effect stemming from the SGL layer. The unidirectional EB anisotropy is due to strongly pinned spins, and the uniaxial rotatable anisotropy to a set of sites where the spin cluster arrangement is movable. A simple superparamagnetic model is developed that incorporates the rotatable anisotropy and accounts for the encountered temperature dependence of the isotropic shift of the FMR field, thus establishing the uniaxial symmetry of the rotatable anisotropy.

## Methods

The preparation of manganese ferrite based NPs and their dispersions (ferrofluids) in an aqueous carrier is achieved by a three steps procedure that has been detailed elsewhere^30,39^. Such a method leads to core–shell NPs with manganese ferrite core surrounded by a maghemite shell (MnFe_2_O_4_@$\gamma$–Fe_2_O_3_) and avoids NPs dissolution in strong acidic medium^31,40^. The local details of their internal structure have been studied by neutron diffration and X-ray absorption Spectroscopy^35^. TEM and HRTEM pictures of the probed NPs are presented in Sect. 1 of “SI”. Recently the morpho-chemical properties of similar core–shell samples synthesized by the same procedure were investigated by using TEM images (STEM mode) with local EDS. The fractions of core and shell phases obtained by chemical analysis match very well with the results of Z-Contrast HAADF images^41,42^. Characteristics of synthesized NPs such as median magnetic diameter entering lognormal size distribution, distribution width, and thickness of the maghemite shell are listed in Table 1. These data are deduced from magnetic measurements and chemical analysis according to Ref.^30^. Besides that, the same analysis renders the average volume fraction $\phi _s/\phi _{p}$ of the maghemite shell inside the NPs and its corresponding layer thickness, Sect. 2 of “SI”. These results are also collected in Table 1.

High-amplitude pulsed measurements are carried out at 1.5 K at the European Magnetic Field Laboratory (EMFL-LNCMI, Toulouse). The data are deduced from the under-field relaxation down to zero field after the field pulse, imposed on dried zero-field-cooled (ZFC) samples prepared by evaporation of water. The results are calibrated with quasistatic magnetization measurements obained at lower field by using a SQUID magnetometer and then the magnetization is normalized by the core volume fraction $\phi _c$ (see Sect. 2 of “SI” for details). The thermal variations of magnetization and field cooled hysteresis loops are obtained on diluted pure water dispersions with a PPMS from Quantum Design operating up to 9 T.

FMR experiments are performed on samples based on NPs, individually dispersed as in Ref.^27^, at a few vol. promilles in a water-glycerine mixture (10:90) to avoid distortion of the absorption lines. The FMR spectra are collected in the temperature interval 3.5–300 K using a Varian E102 spectrometer (Oxford-Instrument cryostat, INSP, UPMC/SU, France) with the probing field of amplitude 800 A/m at frequency 9.26 GHz (X band). In liquid dispersions (ferrofluids), the NPs are mechanically free, so that their easy axes orient themselves in compliance with the applied field. By freezing under zero field, the fluid carrier of a ferrofluid, one fixes the isotropic distribution of the particle axes. If freezing is performed under a constant field ${{\varvec{H}}}_f$, then the NP easy axes texture is quenched possessing the degree of orientation that depends on the field strength $H_f$ and its direction, on the freezing temperature $T_f$ of the fluid dispersion, and on the anisotropy energy as well as on the nature and size of the NPs. For FMR tests, both field-cooled (FC) (implying also orientational texturing in the present work), and ZFC (non-textured) samples are investigated. Here one has to distinguish between *field-frozen* and *field-cooled* situations. The first means that the sample, being initially in liquid state, is then cooled down under field ${\varvec{H}}_f$ to the freezing temperature $T_f$ of the solvent where the sample becomes solid. This procedure fixes the orientational texture that has formed in the sample under ${\varvec{H}}_f$ at $T_f$; subsequently, the freezing field can be removed. The field-cooled (FC) protocol proper is conventional: a sample, already solidified, is cooled down to low temperature (a few Kelvin) under field ${\varvec{H}}_{cool}$ and after that subjected to measurements, e.g., FMR or hysteresis magnetization loops. Evidently, this protocol could be applied to a ferrofluid sample frozen either under zero field (i.e., non textured) or in the presence of some ${\varvec{H}}_f$ (textured). Moreover, the directions of ${\varvec{H}}_{cool}$ and ${\varvec{H}}_f$ are not necessarily the same. A special situation occurs when the FC protocol is applied to a sample that is initially in liquid state. Being subjected to ${\varvec{H}}_{cool}$ from the very beginning, it would solidify at $T_f$ with the orientational texture imposed by the applied field at that point. Further cooling would mean the FC protocol applied to a textured sample for which ${\varvec{H}}_{cool}$ and ${\varvec{H}}_f$ coincide.

## Supplementary information

Supplementary information.

## References

[1] Meiklejohn, W. H. & Bean, C. P. New magnetic anisotropy. *Phys. Rev.* **102**, 1413–1414 (1956).

[2] Phan, M. *et al.* Exchange bias effects in iron oxide-based nanoparticle systems. *Nanomaterials* **6**, 221 (2016). 28335349 10.3390/nano6110221 PMC5245749

[3] Menéndez, E. *et al*. Interdependence between training and magnetization reversal in granular Co-CoO exchange bias systems. *Phys. Rev. B* **89**, 14407 (2014).

[4] Das, R. *et al.* Magnetic anisotropy and switching behavior of Fe_3_O_4_/CoFe_2_O_4_ core/shell nanoparticles. *J. Electron. Mater.* **48**, 1461–1467 (2019).

[5] Flores-Martinez, N. *et al.* On the first evidence of exchange-bias feature in magnetically contrasted consolidates made from CoFe_2_O_4_–CoO core–shell nanoparticles. *Sci. Rep.* **9**, 19468 (2019). 31857610 10.1038/s41598-019-55649-y PMC6923415

[6] Kons, C. *et al.* Investigating spin coupling across a three-dimensional interface in core/shell magnetic nanoparticles. *Phys. Rev. Mater.* **4**, 034408 (2020).

[7] Nogués, J. & Shuller, I. Exchange bias. *J. Magn. Magn. Mater.* **192**, 203–232 (1999).

[8] Skumryev, V. *et al.* Beating the superparamagnetic limit with exchange-bias. *Nature* **423**, 850 (2003). 12815426 10.1038/nature01687

[9] Nogués, J. *et al.* Exchange bias in nanostructures. *Phys. Rep.* **422**, 65–117 (2005).

[10] Martínez, B., Obradors, X., Balcells, L., Rouanet, A. & Monty, C. Low temperature surface spin-glass transition in g-Fe2O_3_ nanoparticles. *Phys. Rev. Lett.* **80**, 181–184 (1998).

[11] Iglesias, O., Labarta, A. & Batlle, X. Exchange bias phenomenology and models of core/shell nanoparticles. *J. Nanosci. Nanotechnol.* **8**, 2761–2780 (2008). 18681014

[12] López-Ortega, J. A., Estrader, M., Salazar-Alvarez, G., Roca, A. G. & Nogués, J. Applications of exchange coupled bi-magnetic hard/soft and soft/hard magnetic core/shell nanoparticles. *Phys. Rep.* **553**, 1–32 (2015).

[13] Khurshid, H. *et al.* Surface spin disorder and exchange-bias in hollow maghemite nanoparticles. *Appl. Phys. Lett.* **101**, 022403 (2012).

[14] Khurshid, H., Phan, M.-H., Mukherjee, P. & Srikanth, H. Tuning exchange bias in Fe/g-Fe_2_O_3_ core–shell nanoparticles: Impacts of interface and surface spins. *Appl. Phys. Lett.* **104**, 072407 (2014).

[15] Khurshid, H. *et al.* Spin-glass-like freezing of inner and outer surface layers in hollow Fe/g-Fe_2_O_3_ nanoparticles. *Sci. Rep.* **5**, 15054 (2015). 26503506 10.1038/srep15054 PMC4621521

[16] Estrader, M. *et al.* Robust antiferromagnetic coupling in hard-soft bi-magnetic core/shell nanoparticles. *Nat. Commun.* **4**, 1–8 (2013). 10.1038/ncomms3960 24343382

[17] Chandra, S. *et al.* Spin dynamics and criteria for onset of exchange bias in superspin glass Fe/g-Fe_2_O_3_ core–shell nanoparticles. *Phys. Rev. B* **86**, 014426 (2012).

[18] Silva, F. G. *et al.* The role of magnetic interactions in exchange bias properties of MnFe_2_O_4_@Fe_2_O_3_ core/shell nanoparticles. *J. Phys. D Appl. Phys.* **46**, 285003 (2013).

[19] Cabreira-Gomes, R. *et al.* Exchange bias of and core/shell nanoparticles. *J. Magn. Magn. Mater.* **368**, 409–414 (2014).

[20] Stiles, M. D. & McMichael, R. D. Coercivity in exchange-bias bilayers. *Phys. Rev. B* **63**, 064405 (2001).

[21] Gomes, R. C. *et al.* Magnetic irreversibility and saturation criteria in ultrasmall bi-magnetic nanoparticles. *J. Alloy. Compd.* **824**, 153646 (2020).

[22] Geshev, J., Pereira, L. G. & Schmidt, J. E. Rotatable anisotropy and coercivity in exchange-bias bilayers. *Phys. Rev. B* **66**, 134432 (2002).

[23] Schafer, D. *et al.* Antiparallel interface coupling evidenced by negative rotatable anisotropy in IrMn/NiFe bilayers. *J. Appl. Phys.* **117**, 215301 (2015).

[24] Prosen, R. J., Holmen, J. O. & Gran, B. E. Rotatable anisotropy in thin permalloy films. *J. Appl. Phys.* **32**, S91–S92 (1961).

[25] Poperechny, I. S., Raikher, Yu. L. & Stepanov, V. I. Superparamagnetic effect in the rotatable anisotropy of nanoparticles and films. *J. Magn. Magn. Mater.* **440**, 192–195 (2017).

[26] Poperechny, I. S. & Raikher, Yu. L. Ferromagnetic resonance in core–shell nanoparticles with multitype exchange anisotropy. *Phys. Rev. B* **98**, 014434 (2018).

[27] Gazeau, F. *et al.* Magnetic resonance of ferrite nanoparticles: Evidence of surface effects. *J. Magn. Magn. Mater.* **186**, 175–187 (1998).

[28] McMichael, R. D., Stiles, M. D., Chen, P. J. & Egelhoff, W. F. Ferromagnetic resonance studies of NiO-coupled thin films of Ni_80_Fe_20_. *Phys. Rev. B* **58**, 8605–8612 (1998).

[29] Shilov, V. P., Raikher, Yu. L., Bacri, J.-C., Gazeau, F. & Perzynski, R. Effect of unidirectional anisotropy on the ferromagnetic resonance in ferrite nanoparticles. *Phys. Rev. B* **60**, 11902–11905 (1999).

[30] Gomes, J. A. *et al.* Synthesis of core–shell ferrite nanoparticles for ferrofluids: Chemical and magnetic analysis. *J. Phys. Chem. C* **112**, 6220–6227 (2008).

[31] Aquino, R. *et al.* Magnetization temperature dependence and freezing of surface spins in magnetic fluids based on ferrite nanoparticles. *Phys. Rev. B* **72**, 184435 (2005).

[32] Coffey, W., Kalmykov, Y. & Titov, S. *Thermal Fluctuations and Relaxation Processes in Nanomagnets* (World Scientific, 2020).

[33] Sousa, E. *et al.* In-field Mössbauer study of disordered surface spins in core/shell ferrite nanoparticles. *J. Appl. Phys.* **106**, 093901 (2009).

[34] Silva, F. G. Magnetic properties, surface disorder and exchange coupling of magnetic nanoparticles. Ph.D. thesis, Cotutelle PhD UnB-Brasilia–Brazil and UPMC-Paris–France (2013).

[35] Martins, F. *et al.* Local structure of core–shell MnFe_2_O_4__−__d_ based nanocrystals: Cation distribution and valence states of manganese ions. *J. Phys. Chem. C* **121**, 8982–8991 (2017).

[36] Del Bianco, L., Fiorani, D., Testa, A. M., Bonetti, E. & Signorini, L. Field-cooling dependence of exchange bias in a granular system of fe nanoparticles embedded in an fe oxide matrix. *Phys. Rev. B* **70**, 052401 (2004).

[37] Nemati, Z. *et al.* From core/shell to hollow Fe/g-Fe_2_O_3_ nanoparticles: Evolution of the magnetic behavior. *Nanotechnol.* **26**(405705), 1–14 (2015). 10.1088/0957-4484/26/40/405705 26376675

[38] Raikher, Yu. L. & Stepanov, V. I. Ferromagnetic resonance in a suspension of single-domain particles. *Phys. Rev. B* **50**, 6250–6259 (1994). 10.1103/physrevb.50.6250 9977000

[39] Tourinho, F. A., Franck, R. & Massart, R. Aqueous ferrofluids based on manganese and cobalt ferrites. *J. Mater. Sci.* **25**, 3249–3254 (1990).

[40] Aquino, R., Tourinho, F., Itri, R., e Lara, M. & Depeyrot, J. Size control of MnFe2O4 nanoparticles in electric double layered magnetic fluid synthesis. *J. Magn. Magn. Mater.* **252**, 23–25 (2002).

[41] Pilati, V. *et al.* Core/shell nanoparticles of non-stoichiometric Zn–Mn and Zn–Co ferrites as thermosensitive heat sources for magnetic fluid hyperthermia. *J. Phys. Chem. C* **122**, 3028–3038 (2018).

[42] Pilati, V., Gomide, G., Cabreira Gomes, R., Goya, G. F. & Depeyrot, J. Colloidal stability and concentration effects on nanoparticles heat delivery for magnetic fluid hyperthermia. *Langmuir*. 10.1021/acs.langmuir.0c03052 (2021). 33443443 10.1021/acs.langmuir.0c03052

