# Correction: Exchange-bias and magnetic anisotropy fields in core–shell ferrite nanoparticles

**DOI:** 10.1038/s41598-025-21913-7

**Published:** 2025-10-01

**Authors:** F. G. Silva, J. Depeyrot, Yu. L. Raikher, V. I. Stepanov, I. S. Poperechny, R. Aquino, G. Ballon, J. Geshev, E. Dubois, R. Perzynski

**Affiliations:** 1 Instituto de Física, Universidade de Brasília, Caixa Postal 04455, Brasília, 70919-970 Brazil; 2 Sorbonne Université, CNRS, PHENIX UMR 8234, 75005 Paris, France; 3 Faculdade UnB Planaltina, Universidade de Brasília, Planaltina (DF), 73345-010 Brazil; 4 Institute of Continuous Media Mechanics, Ural Branch of RAS, Perm, 614068 Russia; 5 Institute of Natural Sciences and Mathematics, Ural Federal University, Ekaterinburg, 620083 Russia; 6 Department of Phase Transitions Physics, Perm State National Research University, Perm, 614990 Russia; 7 CNRS-LNCMI, 31400 Toulouse, France; 8 Instituto de Fisica, UFRGS, Porto Alegre, RS 91501-970 Brazil

Correction to: *Scientific Reports* 10.1038/s41598-021-84843-0, published online 09 March 2021

The original version of this Article contains errors in Table 1, where the values of the surface anisotropy constant $K_{s}$ (J/m^2^) are incorrectly stated as 10^5^ J/m^2^ for the Samples S1, S2 and S3, respectively. The correct and incorrect values appear below.

Incorrect:

**Table:** 

Sample	$K_{s}$ (J/m^2^)
S1	$2.5 \times 10^{5}$
S2	$3.0 \times 10^{5}$
S3	$3.6 \times 10^{5}$

Correct:

**Table:** 

Sample	$K_{s}$ (J/m^2^)
S1	$2.5 \times 10^{-5}$
S2	$3.0 \times 10^{-5}$
S3	$3.6 \times 10^{-5}$

