# Supplementary material for: Exchange-bias and magnetic anisotropy fields in core–shell ferrite nanoparticles
Source: Sci Rep. 2021 Mar 9;11:5474. doi: 10.1038/s41598-021-84843-0 (PMC7970917; doi:10.1038/s41598-021-84843-0)
Supplement: Supplementary file 1 — Supplementary information. [file 41598_2021_84843_MOESM1_ESM.pdf]

SUPPLEMENTARY INFORMATION
Exchange-bias and magnetic anisotropy fields in core-shell ferrite nanoparticles

F. G. Silva[1,2,3*], J. Depeyrot[1], Yu. L. Raikher[4,5], V. I. Stepanov[4], I. S. Poperechny[4,6], R. Aquino[3], G. Ballon[7], J. Geshev[8], E. Dubois[2], and R. Perzynski[2]

[1]Instituto de Física, Universidade de Brasília, Caixa Postal 04455, 70919-970, Brasília, Brazil
[2]Sorbonne Université, CNRS, PHENIX UMR 8234, F-75005, Paris, France
[3]Faculdade UnB Planaltina, Universidade de Brasília, 73345-010, Planaltina (DF), Brazil
[4]Institute of Continuous Media Mechanics, Ural Branch of RAS, Perm, 614068, Russia
[5]Ural Federal University, Ekaterinburg, 620083, Russia
[6]Department of Phase Transitions Physics, Perm State National Research University, Perm, 614990, Russia
[7]CNRS – LNCMI, 31400 Toulouse, France
[8]Instituto de Fisica, UFRGS, Porto Alegre, 91501-970 Rio Grande do Sul, Brazil
[*]franciscarlos@fis.unb.br

1 Analysis of the nanoparticles by TEM and HRTEM

Typical Transmission Electron Microscopy (TEM) images, together wih high-resolution (HRTEM) ones of a single nanoparticle, are presented in Fig. S1 for samples S1 and S3. These TEM images were obtained at Sorbonne Université with a JEOL JEM-100 CX II microscope, while a JEOL JEM-2010 was used for HRTEM.

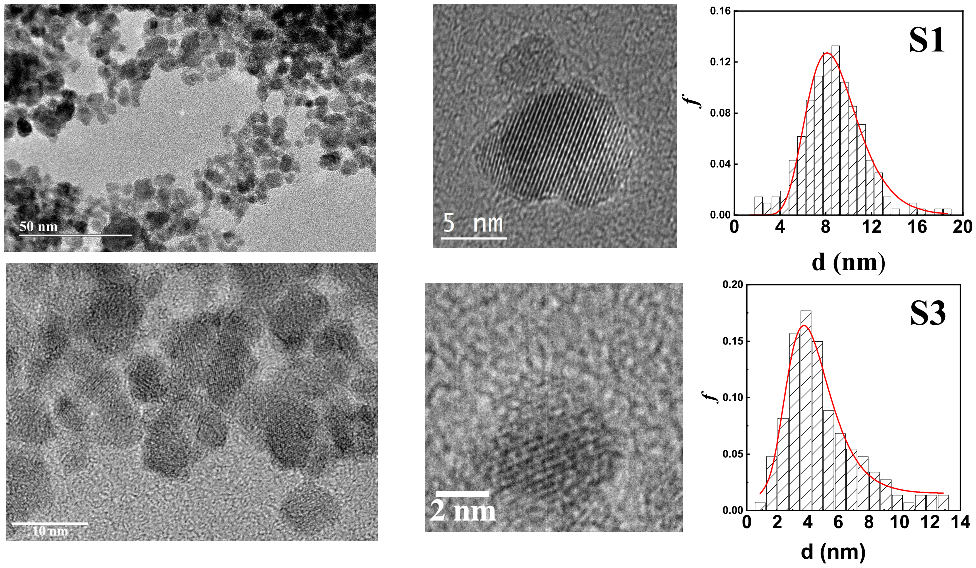

Figure S1. TEM images and High-Resolution TEM ones of single CS-NPs, together with the corresponding normalized histograms of the NP size distribution deduced from TEM measurements; (top) sample S1, (bottom) sample S3.

The histograms were realized on a 200-NPs sampling of micrographs obtained with a dilute sample after evaporation of the liquid carrier. The size distribution is well accounted for by a log-normal law. Fitting of the histograms yields the median diameter (d_0^{TEM}) and polydispersity index (s^{TEM}) which are presented in table S1 of SI for the three samples S1, S2 and S3. They are close to the corresponding magnetic values of Table 1 of the main text.

More details are given in Refs. 1,2 on the morpho-chemical properties of core-shell nanoparticles, similar to these ones

Table S1. Log-normal distribution of diameters deduced from TEM analysis for the different NPs samples; d_0^{TEM} is the median diameter and s^{TEM} the polydispersity index

Sample	d_0^{TEM} (nm)	s^{TEM}
S1	8,0	0.25
S2	3.3	0.3
S3	2.8	0.3

and synthesized by the same procedure. These morpho-chemical properties were investigated by using TEM images (STEM mode) with local EDS. The fractions of core and shell phases obtained by chemical analysis match very well with the results of Z-Contrast HAADF images.

2 Field and temperature dependencies of NPs magnetization - Evaluation of core and shell contributions

The NPs ferrite structure is probed by X-ray diffraction in LNLS-Campinas-Brazil (see Refs. 3 and 4). The molar fractions of Mn and Fe ions in the nanoparticles are determined by ICP-AES (Inductively Coupled Plasma Atomic Emission Spectroscopy). As shown in Ref. 5, it allows to deduce:

- the molar fraction of divalent metal in the core-shell NPs,
- the respective volume proportions ϕ_c and ϕ_{sh} of core and shell inside the nanoparticles,
- the total NPs volume fraction Φ in a liquid dispersion.

The internal density ρ_{NP} of the NPs is deduced from the slope of the density ρ of aqueous dispersions as a function of the total NPs volume fraction Φ, see Ref. 5. SQUID or VSM magnetization measurements are obtained in emu/g under careful weighting of the probed samples. *In liquid dispersions*, magnetization is then easily transformed in emu/cm^3 (i.e., kA/m) by multiplying by ρ; division by Φ yields the averaged magnetization $\overline{M}_p$ of the particle. *In carefully dried powders*, the same conversion can be done when the internal density of the NPs is known. If the powder magnetization, measured in emu/g, is multiplied by ρ_{NP}, one obtains the average particle magnetization in the powder, that may differ from the value deduced in liquid dispersion due to the magnetic interparticle interactions. Very high field measurements are performed at LNCMI-Grenoble on powder samples using the compensated coil technique. The samples are submitted at T =1.5 K, to a short pulse of maximal magnetic field $\mu_0 H = 52$ T, with typical times ~ 5 ms for rising and ~ 300 ms for relaxation. Because of preliminary under-field tests, here only the relaxation part of the measurement is comparable to a standard hysteresis magnetization loop. The relaxing magnetization is then normalized to more standard SQUID measurements of magnetization loops. It is normalized to the branch relaxing from 7 T obtained with the same sample (courtesy of A. Sulpice in CRETA-Grenoble-France) as illustrated by Fig. S2 for sample S3.

In the framework of the simple hypothesis that magnetization contributions from core and shell to the global magnetization imparted by NPs to the sample are additive ($\overline{M} = \overline{M}_c\, \phi_c + \overline{M}_{sh}\, \phi_{sh}$), the overall magnetization $\overline{M}_p$ of a particle can be written as:

$$\overline{M}_p = \overline{M} / \left(\phi_c + \phi_{sh}\right). \tag{1}$$

The shell magnetization is then deduced as

$$\overline{M}_{sh} = \overline{M}_p^{(\mathrm{exp})} - \frac{\phi_c}{\phi_{sh}} \left[\overline{M}_c^{(\mathrm{calc})} - \overline{M}_p^{(\mathrm{exp})}\right], \tag{2}$$

where $\overline{M}_p^{(\mathrm{exp})}$ is the measured magnetization $\overline{M}_p$ of the particle assembly.

We calculate the temperature and field dependencies of the core magnetization taking into account the NPs polydispersity, adapting the classical method of Ref. 6. For that, we consider an assembly of noninteracting uniaxial single-domain NPs

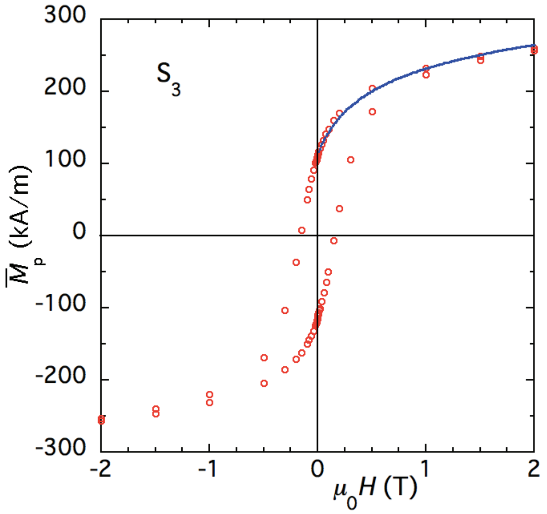

Figure S2. Calibration at low fields of the data obtained from high field relaxation for sample S3 (appearing here as a full line, blue online) to the SQUID ZFC magnetization loop $\overline{M}_p$ (open red symbols).

assuming them as just the cores (no contribution from the shell) frozen in the absence of external field, so that the orientational distribution of the easy axes is isotropic. This system is maintained at temperature T and subjected to an external field $\boldsymbol{H}$. Under assumption that the core diameters obey lognormal law $f(z,s)$, the average magnetization $\overline{M}_c^{(calc)}$ of such a system is

$$\overline{M}_c^{(calc)} = M_c(T)\,\frac{\int dz z^3 f(z,s)\,Q(\xi_0 z^3, \sigma_0 z^2)}{\int dz z^3 f(z,s)}, \tag{3}$$

where $z = d/d_0$ and $M_c(T)$ is the saturation magnetization of the core at temperature T. Function Q in (3) is the average cosine of the angle which the magnetic moment $\boldsymbol{\mu}$ of the particle core makes with the applied field $\boldsymbol{H}$:

$$Q(\xi,\sigma) = \langle(\boldsymbol{eh})\rangle = \frac{1}{4\pi}\int d\boldsymbol{n}\frac{1}{Z}\int d\boldsymbol{e}\,(\boldsymbol{eh})\exp[\xi(\boldsymbol{eh}) + \sigma(\boldsymbol{en})^2]; \tag{4}$$

here $\boldsymbol{e} = \boldsymbol{\mu}/\mu$, $\boldsymbol{h} = \boldsymbol{H}/H$ and $\boldsymbol{n}$ are unit vectors of the magnetic moment of the core, external field and particle easy axis, respectively. The other notations are: $\xi = \mu_0\mu H/kT$ with μ_0 being the vacuum permeability, $\mu = \pi M_c d^3/6$ the magnetic moment of the core and k Boltzmann constant. The anisotropy parameter $\sigma = E_\mathrm{A}/kT$ in Eq. (4) is defined with respect to the uniaxial surface anisotropy energy E_A that is supposed to scale as d^2, as found in Ref.[7]. Function $Z(\xi,\sigma,\boldsymbol{n})$ is the partition integral for a particle core with a given direction of the easy axis. The details of integration over $\boldsymbol{e}$ in Eq. (4) are given in Ref.[6] and in section 2.

In the case of dilute dispersions, the temperature variation of the core magnetization M_c^{calc} is calculated from Eq. (3) with $M_c(T)$ approximated by a modified Bloch law[8]. In order to obtain the field variation of the magnetization (powders), we replace in a first approximation $\overline{M}_c$ at 1.5 K which is unknown, by the value $M_c(0\,\mathrm{K})$ deduced at 5 T in the dilute dispersion from the adjustment of the core contribution by (3) (see Fig 1b of the main text). These values are very close to those of Ref.[8]. The obtained $\overline{M}_{sh}$ contribution is then only meaningful in strong fields: at least, above 5 T. In the future, the $\overline{M}_c(H)$ contribution at 1.5 K will have to be modeled to improve evaluation of $\overline{M}_{sh}(H)$ in low fields.

3 Magnetization of an assembly of superparamagnetic particles with randomly oriented easy axes

For an assembly of nanoparticles, whose axes are distributed at random at a given temperature T under the field $\boldsymbol{H}$, the expression for mean cosine of the angle between the magnetic moment and the applied field, derived in Ref. 6 is given by Eq. 4).

The partition function Z of a particle with a given direction of $\boldsymbol{n}$ is

$$Z(\xi,\sigma,\boldsymbol{n}) = \int d\boldsymbol{e}\exp\left[\xi(\boldsymbol{eh}) + \sigma(\boldsymbol{en})^2\right]. \tag{5}$$

To calculate integrals over $\boldsymbol{e}$ in Eq. (5), we present a single-particle Boltzmann factor as

$$\exp\left[\xi(\boldsymbol{eh})+\sigma(\boldsymbol{en})^2\right]=\exp\left[\xi(\boldsymbol{eh})\right]\cdot\exp\left[\sigma(\boldsymbol{en})^2\right]. \tag{6}$$

For substitution in the right-hand part of Eq. (5), the field-dependent term is expanded in Legendre polynomial series as

$$\exp\left[\xi(\boldsymbol{eh})\right]=4\pi\frac{\sinh\xi}{\xi}\sum_{l=0}^{\infty}\frac{2l+1}{2}L_l(\xi)P_l(\boldsymbol{eh}), \tag{7}$$

where $\{L_l\}$ is the set of Langevin functions

$$L_l(\xi)=\frac{\xi}{4\pi\sinh\xi}\int d\boldsymbol{e}\,P_l(\boldsymbol{eh})\exp\left[\xi(\boldsymbol{eh})\right] \tag{8}$$

beginning with $L_0=1$, so that $L_1=\coth\xi-1/\xi$ is the conventional Langevin function. The anisotropy-induced term in (5) expands in a similar way:

$$\exp\left[\sigma(\boldsymbol{en})^2\right]=4\pi R(\sigma)\sum_{j=0}^{\infty}\frac{2j+1}{2}S_j(\sigma)P_j(\boldsymbol{en}), \tag{9}$$

where

$$S_j(\sigma)=\frac{1}{4\pi R(\sigma)}\int d\boldsymbol{e}\,P_j(\boldsymbol{en})\exp\left[\sigma(\boldsymbol{en})^2\right], \tag{10}$$

and

$$R(\sigma)=\int_0^1\exp(\sigma x^2)\,dx. \tag{11}$$

As seen from definition (10) of functions $S_j(\sigma)$, all the odd-index terms vanish. At $j=2$ one gets the factor S_2 entering Eqs. (2) and (3) of the main text. Substituting Eqs. (7), (8) and (9) in (6), one gets

$$\exp\left[\xi(\boldsymbol{eh})+\sigma(\boldsymbol{en})^2\right]=16\pi^2R(\sigma)\times\frac{\sinh\xi}{\xi}\sum_{j=0}^{\infty}\frac{2j+1}{2}S_j(\sigma)P_j(\boldsymbol{en})\times\sum_{l=0}^{\infty}\frac{2l+1}{2}L_l(\xi)P_l(\boldsymbol{eh}). \tag{12}$$

With the aid of Eq.(12) and well-known transformation relations for Legendre polynomials

$$\int d\boldsymbol{e}P_l(\boldsymbol{en})P_j(\boldsymbol{eh})=\frac{4\pi}{2l+1}\,\delta_{lj}\,P_l(\boldsymbol{nh}),\qquad \cos(x)P_l(x)=\frac{1}{2l+1}\left[lP_{l-1}(x)+(l+1)P_{l+1}(x)\right], \tag{13}$$

integrations over $\boldsymbol{e}$ and then in (5) and then in (3) are done analytically yielding the sought for function in the form

$$Q(\xi,\sigma)=\int d\boldsymbol{n}\,\frac{\sum_{l=0}^{\infty}\left[lL_{l-1}(\xi)+(l+1)L_{l+1}(\xi)\right]S_l(\sigma)P_l(\boldsymbol{nh})}{\sum_{l=0}^{\infty}(2l+1)L_l(\xi)S_l(\sigma)P_l(\boldsymbol{nh})}. \tag{14}$$

4 Superparamagnetic “thawing” of single-particle rotatable anisotropy

The model single-domain particle is characterized by its magnetic moment vector $\boldsymbol{\mu}$ with a constant length μ but variable orientation $\boldsymbol{e}=\boldsymbol{\mu}/\mu$. The motion of vector $\boldsymbol{e}$ under a field, which combines the thermal rotary diffusion, Larmor precession and intrinsic magnetic relaxation, is described by the Landau-Lifshitz-Gilbert equation into which a white noise is added to allow for thermal fluctuations. The orientation-dependent energy function that accounts for coexisting uniaxial core, unidirectional exchange-bias (EB), and uniaxial rotatable anisotropy (RA) is written in the form

$$E=-\mu_0\mu H(\boldsymbol{eh})-E_{\rm A}(\boldsymbol{en})^2-E_{\rm EB}(\boldsymbol{eh}_c)-E_{\rm RA}(\boldsymbol{eh})^2, \tag{15}$$

where $\boldsymbol{h}=\boldsymbol{H}/H$ is the direction of magnetizing field, $\boldsymbol{h}_c=\boldsymbol{H}_{cool}/H_{cool}$ the direction of the cooling field (if any) and $\boldsymbol{n}$ unit vector of the anisotropy axis whose direction is fixed with respect to the particle body. Note that we neglect the bulk anisotropy of the core and take into account only its uniaxial surface anisotropy (of the Aharoni type[9]) presenting the corresponding reference energy as $E_{\rm A}=\pi K_s d^2$, where K_s is the surface density of that energy.

The regular part of the acting field comes out as $\boldsymbol{H}_{act} = -(\mu_0\mu)^{-1}\partial E/\partial \boldsymbol{e}$ with energy E from (15):

$$\boldsymbol{H}_{act} = H\boldsymbol{h} + H_{\rm A}^{(0)}(\boldsymbol{en})\boldsymbol{n} + H_{\rm EB}^{(0)}\boldsymbol{h}_c + H_{\rm RA}^{(0)}(\boldsymbol{eh})\boldsymbol{h}, \tag{16}$$

where, $H_{\rm A}^{(0)} = \frac{2E_{\rm A}^{(0)}}{\mu_0\mu}$, $H_{\rm EB}^{(0)} = \frac{E_{\rm EB}^{(0)}}{\mu_0\mu}$, $H_{\rm RA}^{(0)} = \frac{2E_{\rm RA}^{(0)}}{\mu_0\mu}$, superscripts denoting the respective values at zero temperature.

The FMR problem for a particle, whose magnetic moment moves in the sum of external and anisotropy fields and is also affected by thermal fluctuations (superparamagnetism), can be formulated and solved in the way similar to that used in Ref. 10. The main assumption there is that the anisotropy energy is smaller than the Zeeman one. Upon doing that and neglecting the precession damping, one arrives at the expression for the resonance field of a particle with a given orientation of axis $\boldsymbol{n}$ in the form

$$H_r(\vartheta) = \frac{\omega}{\gamma} - H_{\rm A}^{(0)}\frac{L_2(\xi_L)}{L_1(\xi_L)}P_2(\cos\vartheta) - H_{\rm EB}^{(0)}\cos\vartheta_c - H_{\rm RA}^{(0)}\frac{L_2(\xi_L)}{L_1(\xi_L)} \tag{17}$$

with $\xi_L = \mu_0\mu\omega/\gamma kT$, $\cos\vartheta = (\boldsymbol{nh})$, $\cos\vartheta_c = (\boldsymbol{hh}_c)$.

The temperature dependence enters Eq. (17) through Langevin functions $L_1(\xi)$ and $L_2 = 1-3L_1(\xi)/\xi$. At low temperatures ($\xi \gg 1$) the ratio L_2/L_1 tends to unity, in the high temperature limit ($\xi \to 0$) it "thaws" approaching zero. The dependence of the FMR field H_r on the angle ϑ between the applied field and the core anisotropy axis is rendered by Legendre polynomials $P_1 = \cos\vartheta$ and $P_2 = \frac{3}{2}\cos^2\vartheta - \frac{1}{2}$.

In the low-temperature limit, for the particles with axes $\boldsymbol{n}$ perfectly oriented along ($\theta = 0^\circ$) and across ($\vartheta = 90^\circ$) the magnetizing field, with $\boldsymbol{n}$ parallel to $\boldsymbol{h}_c$ ($\vartheta = \vartheta_c$), Eq. 17 yields

$$H_r(0^\circ) = \frac{\omega}{\gamma} - H_{\rm A}^{(0)} - H_{\rm EB}^{(0)} - H_{\rm RA}^{(0)}, \tag{18}$$

$$H_r(90^\circ) = \frac{\omega}{\gamma} + \tfrac{1}{2}H_{\rm A}^{(0)} - H_{\rm RA}^{(0)}, \tag{19}$$

respectively. Then, the difference of Eqs. (18) and (19) in a simple way connects the strength of the surface anisotropy field $H_{\rm A}$ with the FMR parameters[11]:

$$H_{\rm A}^{(0)} = \tfrac{2}{3}\left[H_r(90^\circ) - H_r(0^\circ) - H_{\rm EB}^{(0)}\right]. \tag{20}$$

On the other hand, the sum of equations (16) renders the RA field in the form

$$H_{\rm RA}^{(0)} = \frac{\omega}{\gamma} - \tfrac{1}{3}\left[2H_r(90^\circ) + H_r(0^\circ) + H_{\rm EB}^{(0)}\right]. \tag{21}$$

In a particle assembly (with EB absent) frozen from a liquid state under a given constant field $\boldsymbol{H}_f$, the distribution of anisotropy axes is fixed and equals the one established by that field at the freezing temperature T_f. Given that, the angle functions in Eq. (17) should be replaced by their averages over the orientational distribution that is the equilibrium one for $\boldsymbol{H}_f$ at T_f. Upon introducing the Langevin argument at the freezing point: $\xi_f = \mu_0\mu H_f/kT_f$, Eq. (17) transforms to

$$H_r(\vartheta_f) = \frac{\omega}{\gamma} - H_{\rm A}^{(0)}\frac{L_2(\xi_L)}{L_1(\xi_L)}S_2(\sigma_f)L_2(\xi_f)P_2(\cos\vartheta_f) - H_{\rm RA}^{(0)}\frac{L_2(\xi_L)}{L_1(\xi_L)}, \tag{22}$$

where factor $L_2(\xi_f)S_2(\sigma_f)$ renders the orientational distribution acquired under field $\boldsymbol{H}_f$ at T_f, and function S_2 is defined in Eq. (3) of the main text. In the presence of EB induced by the FC treatment an additional term,

$$-H_{\rm EB}\cos\vartheta_c, \quad \text{where} \quad \cos\vartheta_c = (\boldsymbol{hh}_c), \tag{23}$$

should be added in the right-hand part of equation (22). If $\boldsymbol{H}_{cool}$ is applied after solidification, then ϑ_f and ϑ_c are, in general, different; if $\boldsymbol{H}_{cool}$ is applied before solidification, i.e., it simultaneously works as $\boldsymbol{H}_f$, then $\vartheta_f = \vartheta_c$. Note that in either of the cases there is no superparamagnetic renormalization of the EB field, the temperature dependence of $H_{\rm EB}$ is entirely defined by the intrinsic properties of the CS interface.

As seen, in an ensemble (without EB) frozen under a field, the axis of the orientational texture is defined by unit vector $\boldsymbol{h}_f$. Assuming that in a FMR measurement such a sample is rotated with respect to the magnetizing field from 0 to 90°, from Eq. (22) one gets the difference of the resonance fields

$$\Delta H_r(90^\circ) = H_r(90^\circ) - H_r(0^\circ) = \frac{18K_s}{\mu_0 M_c d}\frac{L_2(\xi_L)}{L_1(\xi_L)}L_2(\xi_f)S_2(\sigma_f). \tag{24}$$

In Eq. (24), the anisotropy field $H_{\mathrm{A}}^{(0)}$ is expressed via the anisotropy energy $E_{\mathrm{A}} = \pi K_s d^2$ and the particle magnetic moment $\mu = (\pi/6) M_c d^3$. Note that in Eqs. (22) and (24) the resonance field values now mean the data taken on a particle assembly, i.e., a macroscopic sample. The only remaining "artificial" feature is that those equations are derived for a set of identical particles whereas the real systems are polydisperse.

In the same monodisperse approximation, the temperature-dependent rotatable anisotropy field is presented via the reference FMR measurement data as

$$H_{\mathrm{RA}}(T) = H_{\mathrm{RA}}^{(0)} \frac{L_2(\xi_L)}{L_1(\xi_L)} = \left[\frac{\omega}{\gamma} - \tfrac{2}{3} H_r(90^\circ) - \tfrac{1}{3} H_r(0^\circ) \right]. \tag{25}$$

In the data adjustments, the polydispersity in diameter is introduced in Eqs. (22) and (25), in the same way as for the calculation of M_c in Eq. (3).

References

1. Pilati, V. *et al.* Core/Shell Nanoparticles of Non-Stoichiometric Zn–Mn and Zn–Co Ferrites as Thermosensitive Heat Sources for Magnetic Fluid Hyperthermia. *J. Phys. Chem. C* **122**, 3028–3038 (2018).
2. Pilati, V., Gomide, G., Cabreira Gomes, R., Goya, G. F. & Depeyrot, J. Colloidal stability and concentration effects on nanoparticles heat delivery for magnetic fluid hyperthermia. *Langmuir* DOI: 10.1021/acs.langmuir.0c03052 (2021).
3. Silva, F. G. *et al.* The role of magnetic interactions in exchange bias properties of $MnFe_2O_4$@Fe_2O_3 core/shell nanoparticles. *J. Phys. D: Appl. Phys.* **46**, 285003 (2013).
4. Martins, F. *et al.* Local structure of core-shell $MnFe_2O_{4+\delta}$ based nanocrystals: Cation distribution and valence states of manganese ions. *J. Phys. Chem. C* **121**, 8982–8991 (2017).
5. Gomes, J. A. *et al.* Synthesis of core-shell ferrite nanoparticles for ferrofluids: chemical and magnetic analysis. *J. Phys. Chem. C* **112**, 6220–6227 (2008).
6. Raikher, Yu. L. The magnetization curve of a textured ferrofluid. *J. Magn. Magn. Mater.* **39**, 11–13 (1983).
7. Shilov, V. P., Raikher, Yu. L., Bacri, J.-C., Gazeau, F. & Perzynski, R. Effect of unidirectional anisotropy on the ferromagnetic resonance in ferrite nanoparticles. *Phys. Rev. B* **60**, 11902–11905 (1999).
8. Aquino, R. *et al.* Magnetization temperature dependence and freezing of surface spins in magnetic fluids based on ferrite nanoparticles. *Phys. Rev. B* **72**, 184435 (2005).
9. Aharoni, A. Surface anisotropy in micromagnetics. *J. Appl. Phys.* **61**, 3302–3304 (1987).
10. Raikher, Yu. L. & Stepanov, V. I. Ferromagnetic resonance in a suspension of single-domain particles. *Phys. Rev. B* **50**, 6250–6259 (1994).
11. Gazeau, F. *et al.* Magnetic resonance of ferrite nanoparticles: evidence of surface effects. *J. Magn. Magn. Mater.* **186**, 175–187 (1998).
